# Relaxation time of brain tissue in the elderly assessed by synthetic MRI

**DOI:** 10.1002/brb3.2449

**Published:** 2021-12-04

**Authors:** Martin Ndengera, Bénédicte M. A. Delattre, Max Scheffler, Karl‐Olof Lövblad, Torstein R. Meling, Maria Isabel Vargas

**Affiliations:** ^1^ Division of Neurosurgery Department of Clinical Neurosciences Geneva University Hospitals Geneva Switzerland; ^2^ Division of Radiology Department of Diagnostics Geneva University Hospitals Geneva Switzerland; ^3^ Division of Neuroradiology Department of Diagnostics Geneva University Hospitals Geneva Switzerland; ^4^ Faculty of Medicine University of Geneva Geneva Switzerland

**Keywords:** brain, elderly, MRI, radiology, relaxation times, synthetic imaging, T1, T2

## Abstract

**Background:**

Synthetic MRI (SyMRI) is a quantitative technique that allows measurements of T1 and T2 relaxation times (RTs). Brain RT evolution across lifespan is well described for the younger population. The aim was to study RTs of brain parenchyma in a healthy geriatric population in order to define the normal value of ***structures*** in this group population. Normal values for geriatric population could help find biomarker for age‐related brain disease.

**Materials and methods:**

Fifty‐four normal‐functioning individuals (22 females, 32 males) with mean age of 83 years (range 56–98) underwent SyMRI. RT values in manually defined ROIs (centrum semiovale, middle cerebellar peduncles, thalamus, and insular cortex) and in segmented whole‐brain components (brain parenchyma, gray matter, white matter, myelin, CSF, and stromal structures) were extracted from the SyMRI segmentation software. Patients' results were combined into the group age. Main ROI‐based and whole‐brain results were compared for the all dataset and for age group results as well.

**Results:**

For white matter, RTs between ROI‐based analyses and whole‐brain results for T2 and for T1 were statistically different and a trend of increasing T1 in centrum semiovale and cerebellar peduncle was observed. For gray matter, thalamic T1 was statistically different from insular T1. A difference was also found between left and right insula (*p* < .0001). T1 RTs of ROI‐based and whole‐brain‐based analyses were statistically different (*p* < .0001). No significant difference in T1 and T2 was found between age groups on ROI‐based analysis, but T1 in centrum semiovale and thalamus increased with age. No statistical difference between age groups was found for the various segmented volumes except for myelin between 65–74 years of age and the 95–105 years of age groups (*p* = .038).

**Conclusions:**

SyMRI is a new tool that allows faster imaging and permits to obtain quantitative T1 and T2. By defining RT values of different brain components of normal‐functioning elderly individuals, this technique may be used as a biomarker for clinical disorders like dementia.

## Graphical abstract

The first paper to describe relaxation values with synthetic imaging of brain tissue in elderly patients. The MRI results may be used to detect abnormal values in dementia disorders such as Alzheimer disease at early stages.

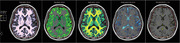

## 1 INTRODUCTION

Synthetic MRI (SyMRI) generates high‐quality qualitative and quantitative MRI images based on different tissue properties. Synthetic images are generated from parametric tissue maps and permits the generation of several differently contrast‐weighted sequences out of one single MRI acquisition. Consequently, this method has a mild time‐saving potential compared to a traditional MRI exam, where several acquisitions are required to obtain sequences that are different in contrast. Most importantly, it is a quantitative technique that provides tissue T1 and T2 relaxation times as well as proton density maps, invariable parameters that depend on the composition of the distinct tissues studied (Hagiwara et al., 2017; West et al., 2012). Fingerprinting another technic using pseudorandomized variation of the sequence parameter and using a dictionary to classify each voxel also gives quantitative and qualitative data for each tissue (Ma et al., 2013).

As emphasized by certain research comparing SyMRI to conventional technics, the time‐saving potential of this technique, in addition to the ability to obtain various contrast imaging from one acquisition, is even more relevant when considering the ability to perform segmentation of the various brain compartments (Hagiwara et al., 2017). Another advantage over current MRI sequences lies in the fact that relaxation times obtained by synthetic sequences are independent of the MRI system used partially removing the B1 inhomogeneity, and may potentially be more accurate and reproducible than those obtained by traditional techniques (European Society of R, 2015; Fujita et al., 2019). This property could increase our capacity to compare data acquired in various centers and ultimately improve the ability to study brain modifications related to aging or progressive disorder like neurodegenerative disease.

The aim of our study was to measure relaxation parameters acquired by SyMRI of the brain in a normal‐functioning geriatric population. Indeed, it is well known that aging of the brain results in alterations of relaxation times that can be measured by MRI (Gracien et al., 2017). However, the relationship between T1 and T2 relaxation times of brain tissue and patient age is not linear (Knight et al., 2016). Unlike in pediatric population, where relaxation times for brain tissues are well documented with SyMRI (Betts et al., 2016; Lewis et al., 2019; Mcallister et al., 2017), no study has so far presented relaxation parameters acquired with this technique in an elderly population.

## 2 MATERIALS AND METHODS

### 2.1 Population

Sixty‐two consecutive individuals without any significant clinical history and with MRI scans considered normal for age were included in our study. Eight individuals were excluded due to artifacts. We did not exclude patients with asymptomatic white matter alterations. Exams of 54 individuals (22 females, 32 males; mean age 83 years; range 56–98 years) were finally analyzed.

The included individuals were divided into five age groups (55–64, 65–74, 75–84, 85–94, and 95–105 years). The number of individuals, age distribution, and sex ratio of every group are shown in Table S1.

### 2.2 Image acquisition

The SyMRI sequence was added to the clinical MRI protocol for elderly patients who underwent brain MRI after a transient neurological focal deficit. 2D axial acquisitions were performed using a 3T MRI scanner (Magnetom Skyra, Siemens Healthineers, Erlangen, Germany). The MRI protocol was composed of T2 turbo spin‐echo, T1 spin‐echo, and fluid‐attenuated inversion recovery (FLAIR) sequences, as well as the SyMRI sequence with the following parameters: FOV = 220 × 172 mm, matrix size = 320 × 188, TE = 23–101 ms, TR = 4790 ms, slice thickness = 4 mm with a 0.4 mm gap between slices, band width = 150 Hz/pixel, parallel imaging with GRAPPA factor = 3, for an acquisition time of 5 min and 27 s. The SyMRI sequence allowed to generate eight images per slice, based on four different TIs and two different TEs.

### 2.3 Image analysis

The conventional and synthetic images of each participant were reviewed by a fully trained neuroradiologist on a clinical workspace station. SyMRI sequences were processed using the SyMRI dedicated software in version 7.3.2 (SyntheticMR, Linköping, Sweden) for both region of interest (ROI)‐based and whole‐brain (WB) analyses, with extraction of relaxation times. Indeed, we performed both analysis in order to compare the ability of the software to segment various brain structures.

### 2.4 ROI‐based assessment

**FIGURE 1:**
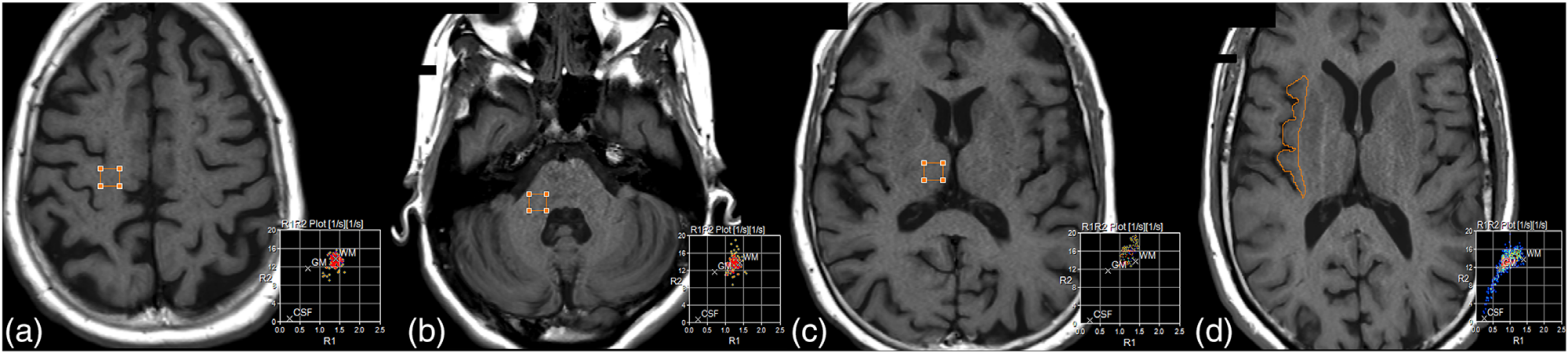
T1 weighted images showing the locations of the region of interest (ROI). For every individual, six square ROIs (8.1 mm x 8.1 mm) were placed on synthetic image series; four in white matter and two in gray matter. The white mater ROIs were placed in the centrum semiovale at the level of the hand‐knob (a) and in the middle cerebellar peduncle (b) on either side. Care was taken to avoid any area containing white mater alteration. The gray matter ROIs were placed in the thalamus (c) at the level of the interventricular foramen on either side. In addition, ROIs were manually drawn on either side to contour the insula as precisely as possible, in order to avoid inclusion of the extreme capsule (d). The right lower part of each slice shows the corresponding R1 (1/T1 in ms^−1^) versus R2 (1/T2 in ms^−1^) plot measured in the voxels of the ROI. The plot shows the R1, R2 for WM, GM, and CSF as a comparison

For each individual, six square ROIs, all of 66 mm^2^ (8.1 mm × 8.1 mm), were placed on the SyMRI images, four in the white matter (WM) and two in the gray matter (GM). All ROI locations were chosen because of their reproducibility. Two white matter ROIs, one on each side, were placed in the centrum semiovale at the level of the omega shaped like precentral gyrus, the so‐called “hand‐knob” (Yousry, 1997). Care was taken to avoid areas marked by any white matter alteration. Two white matter ROIs were placed in the middle cerebellar peduncle on either side in order to analyze both supra‐ and infratentorial white mater. For gray matter, two ROIs were manually drawn in order to contour both insulae as precisely as possible, with the exclusion of white matter in the extreme capsule. To obtain subcortical gray matter signal, two additional ROIs were placed in both thalami at the level of the interventricular foramen. Figure 1 summarizes all ROI locations, as shown on one side.

Means, as well as standard deviations, for T1 and T2 relaxation times of pixels contained within the ROIs were extracted. For each ROI, we also extracted the calculated normalized proton density (PD) in percent.

### 2.5 Whole‐brain assessment

For every individual, the intracranial volume was segmented according to the various structures using the dedicated SyMRI tool. The software created the following outputs: white matter, gray matter, myelin, CSF, and NON (i.e., non‐white matter, non‐gray matter, and non‐CSF). Myelin segmentation comes from a compartmental model calculation with four compartments each of them having specific R1, R2, and PD (Hagiwara et al., 2017; Warntjes et al., 2016).

**FIGURE 2:**
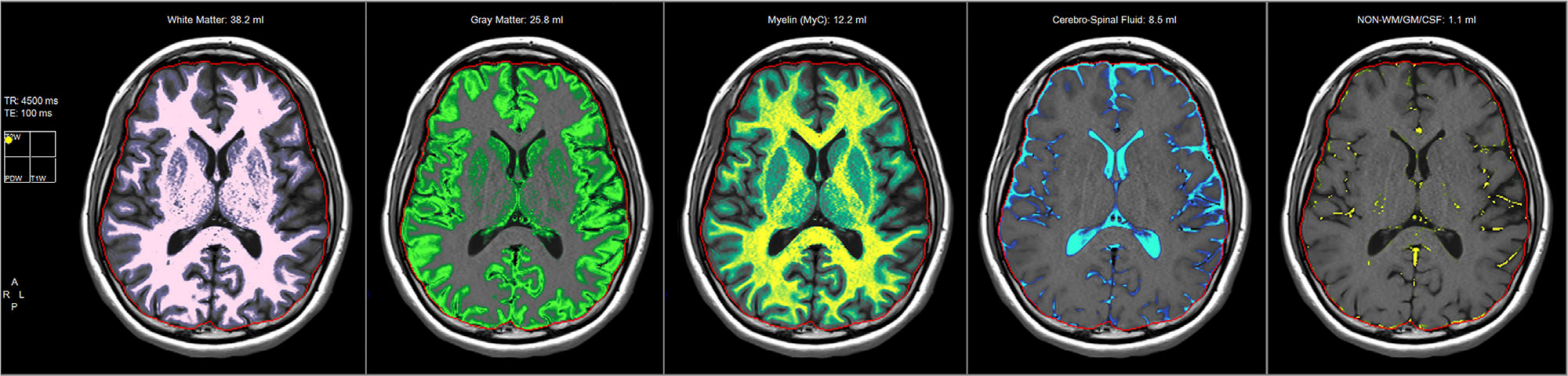
For every individual, the intracranial volume was segmented using a dedicated Synthetic MRI tool. The software created the following segmentations: white matter, gray matter, myelin, CSF, and NON (i.e., non‐white matter, non‐gray matter, and non‐CSF)

In the software, segmentation of the various compartments is achieved using predefined clusters of specific T1, T2, and PD values intended to represent white matter, gray matter, and CSF. Tissue that does not correspond to any of the predefined values is labeled as “NON.” The software displays the segmentation to the user in a way that allows verification of the quality of the segmentation (Figure 2).

For every individual segmentation, two types of data were extracted; volumetric and relaxation parameters. The volume of tissue associated with one segmentation was expressed in milliliters (ml). The alteration containing white matter tissue was excluded from the volumetric quantification and classified as “NON” by the software. Means and standard deviations of T1 and T2 relaxation times associated with the different compartments were recorded. Lastly, we extracted the normalized PD, indicated in percent.

To compare ROIs and WB results, we used the centrum semiovale ROIs for comparison with the white matter WB results, and the insula ROIs for comparison with the gray mater WB results.

### 2.6 Statistics

Statistical analyses were performed using Prism 7.0 (GraphPad Software, San Diego, USA). To compare the means of two ROIs and for comparison of ROI mean results and the mean results acquired by whole‐brain analysis, we used the Wilcoxon paired nonparametric test. The same test was used when comparing WB and ROI‐based results by age and to compare two‐by‐two the age groups of the two categories. When comparing the means between the various groups of ages, we used ANOVA (Kruskal–Wallis test) with a Dunn's post hoc test. The correlation between age and volumetric data was calculated using Spearman correlation.

### 2.7 Ethics

The study was approved by the competent ethics commission (CCER 2016−1821). Patient informed consent was waived.

## 3 RESULTS

### 3.1 ROI‐based analysis

As shown in (Table S2), white matter ROIs for all individuals had a mean T1 relaxation time of 900.4 ms with a standard deviation (*σ*) of 63.3 ms. The T2 was 81.84 ms (*σ*: 5.0 ms), and the PD 67.79 pu (*σ*: 2.2 pu; pu = proton concentration in water). Gray matter ROIs had a mean T1 of 1120 ms with a standard deviation of 69 ms. The T2 was 74.69 ms (*σ*: 5 ms), and the PD 77.89 pu (*σ*: 2 pu). The T1 and T2 relaxation times of gray and white matter were statistically different (*p* < .0001). The T1 and T2 of the thalamus (1031 and 69.34 ms, respectively) were significantly different from those of the insula (T1, 1209 ms; T2, 80.03 ms) (*p* < .0001).

Analysis of the white matter ROIs also showed statistically significant differences. The relaxation times of the centrum semiovale (T1, 936.2 ms; T2, 107.7 ms) were statistically different from those of the cerebellar peduncle (T1, 864.6 ms; T2, 123.2 ms) (*p* < .0001 and *p* = .0024, respectively).

The comparisons between left and right ROIs did not show significant difference for white matter T1 (*p* = .1) or T2 (*p* = .9). For gray matter, we found a small difference between the right and left thalamus in mean T2 (right, 70 ms; left, 68.7 ms; *p* = .036) and a difference between right and left insula in T1 (right, 1238 ms; left, 1181 ms; *p* < .0001) and in T2 (left, 78.7 ms; right, 81.35 ms; *p* = .0047).

### 3.2 ROI‐based analysis by age

**FIGURE 3:**
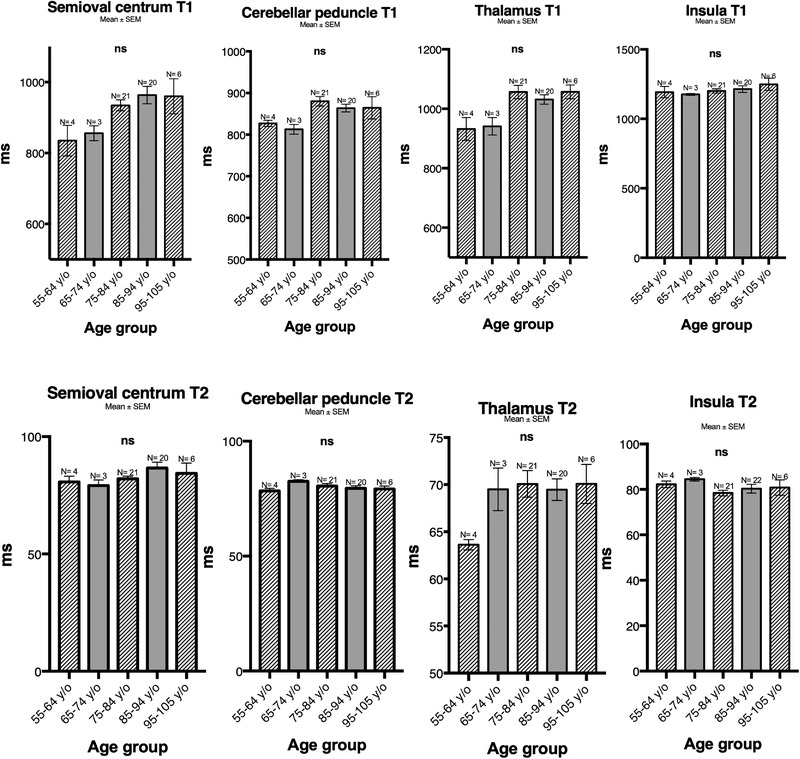
Mean ±standard error of mean (SEM) relaxation times in milliseconds (ms) (*y*‐axis) by different age groups (*x*‐axis) of the centrum semiovale, cerebellar peduncle, thalamus, and insula from region of interest (ROI)‐based analyses of T1 (upper graphs) and T2 (lower graphs) weighted images. No significant difference in T1 or T2 was found between the various age groups

As shown in Figure 3, when pooled according to age groups, no significant difference in T1 and T2 was found between the groups. However, a tendency of increasing T1 over age could be seen for the centrum semiovale and the thalamus.

### 3.3 Whole‐brain analysis

Using whole‐brain automated segmentation, the mean T1 for GM was 1747 ms (*σ* = 72.2 ms) and the mean T2 104.4 ms (*σ* = 4.2 ms). In the WM, a mean T1 of 904.4 ms (*σ* = 43.5 ms) was found, and a mean T2 of 69.8 ms (*σ* = 2.4 ms). The mean T1 for myelin was 902 ms (*σ* = 47.5 ms), and the mean T2 71.7 ms (*σ* = 3.1 ms). For CSF, the mean T1 was 3742 ms (*σ* = 127.3 ms), and the mean T2 599.2 ms (*σ* = 57.2 ms).

### 3.4 Whole‐brain analysis by age

**FIGURE 4:**
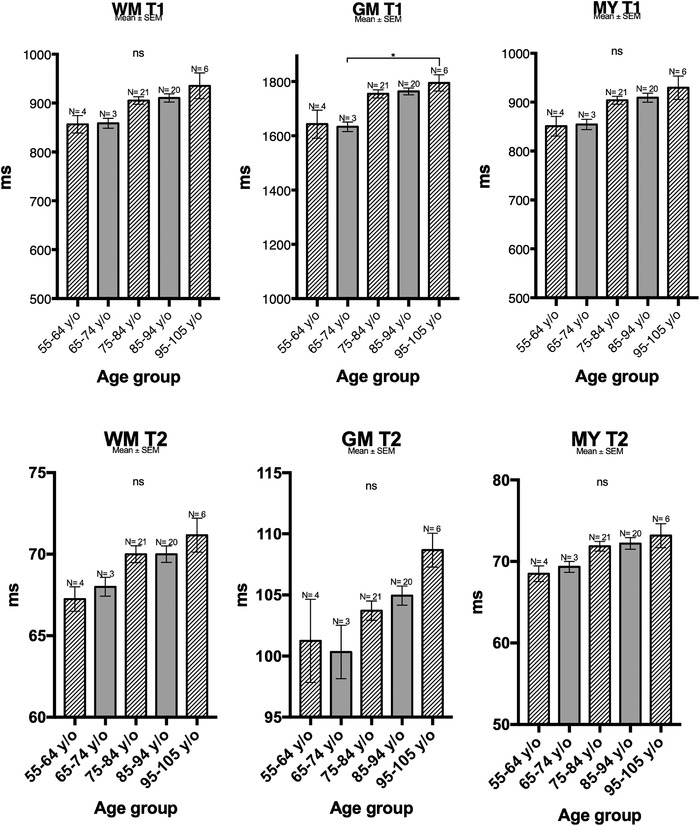
Mean ±standard error of mean (SEM) relaxation times in milliseconds (ms) (*y*‐axis) by different age groups (*x*‐axis) of white matter (WM), grey matter (GM) and myelin (MY) from whole brain (WB)‐based analyses of T1 (upper graphs) and T2 (lower graphs) weighted images. A trend of increasing T2 with increasing age is seen for both WM and GM in T2 and in T1 to a lesser extent, but no statistically significant difference is seen except in GM T1 between patients from the 65 to 74 year age groups and those from 95 to 105 year age group

As shown by Figure S4, the spearman correlation between age and RT is low but significant for WM, GM, brain, and myelin in T1 and in T2. When split up by age group, the results of the whole‐brain analyses showed no statistical difference between the different groups except for T1 in the GM, where a barely significant difference (*p* = .047) between individuals of around 60 and 100 years of age (Figure 4, Figure S2) was found. However, we could see a clear trend of longer T2 times with increasing age for both WM and GM. The same trend could be observed for T1, but to a lesser extent.

**FIGURE 5:**
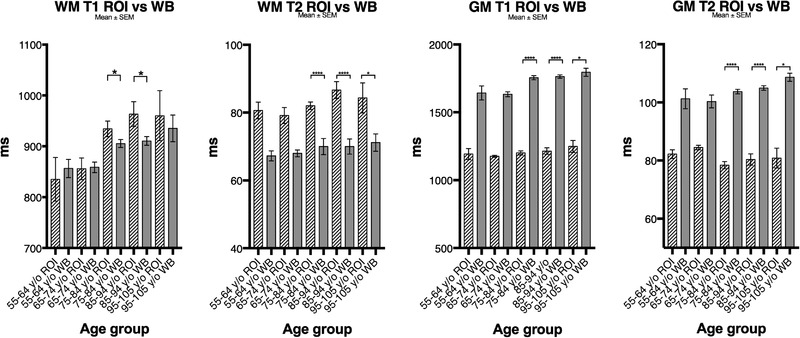
Mean ±standard error of mean (SEM) relaxation times in milliseconds (ms) (*y*‐axis) by different age groups (*x*‐axis) of white matter (WM) and grey matter (GM) from region of interest (ROI)‐based and whole‐brain (WB)‐based analyses of T1 and T2 weighted images. Statistically significant differences are seen in T1 and T2 for both GM and WM between ROI‐based and WB‐based analyses of the oldest groups of patients

When whole‐brain auto‐segmented results were compared to the ROI‐based results, we found a highly significant difference for T1 and T2 in GM and for T2 in WM for the oldest age groups (Figure 5).

### 3.5 Volumetric analysis

No statistical difference in terms of volume between age groups was found for the various segmented volumes except for myelin between 65–74 years of age and the 95–105 years of age groups (*p* = .038) (Figure S1). We nevertheless observed a tendency of WM and myelin to decrease with age and CSF to increase with age. These trends were well visualized by the correlations between volume and age (*r* = −0.31, *p* = .02 for WM; *r* = −0.32, *p* = .01 for myelin; *r* = 0.36, *p* = .007 for CSF). The correlations were stronger when analyzed in terms of proportions of total intracranial volume (*r* = 0.38, *p* = .0038 for WM; *r* = −0.41, *p* = .002 for myelin; *r* = 0.48, *p* = .0002 for CSF) (Figure S1)

## 4 DISCUSSION

A quantitative analysis using SyMRI of the whole brain and selected intracerebral structures was performed in order to define standard values of T1 and T2 relaxation times and volumes of different compartments in a population of elderly individuals.

### 4.1 White matter

For white matter, we found statistical differences in relaxation times between ROI‐based analyses and whole‐brain results for T2 and for T1. The pooled analyses according to age groups did not identify any statistically significant differences between the various groups concerning ROI‐based results. However, a trend toward increasing T1 of the centrum semiovale and the cerebellar peduncle was clearly observed. No difference was found between left‐ and right‐sided ROIs neither in T1 nor T2.

We did find a significant difference between the centrum semiovale and the cerebellar peduncle. This finding is not surprising, knowing that white matter is very heterogeneous. Indeed, histological heterogeneity of white matter is well known in terms of fiber orientation and density, but it has already been shown that white mater is also heterogeneous with respect to relaxation times in various regions (Agartz et al., 1991; Bojorquez et al., 2017; Cho et al., 1997).

The T1 values found in cerebellar peduncles and the centrum semiovale were higher than some of those found by Cho et al. (1997). That group found different values depending on the exact measuring point, illustrating the heterogeneity also seen in our data. This heterogeneity could partially explain the differences we saw between ROI‐based and whole‐brain analyses. On the other hand, the differences between our values and those of Cho et al. (1997) could be explained by age; the mean age of their population was 26.5 years whereas ours was 83 years. Indeed, in the Cho et al. (1997) study, T1 values clearly increased from the age of 40 years onward. We did not find a statistically significant trend between age and WM T1 in our study population.

Preibisch and Deichmann (2009) found relaxation time values more in line with ours. Importantly, in addition to age and other biological factors, relaxation times were also influenced by technical parameters and measurement technique in general, a finding that has been reported in other studies (Bojorquez et al., 2017). However, the SyMRI technique used in our study has previously been validated for T1 and T2 times ranging from 200 to 2000 ms and 40 to 400 ms, respectively (AB, 2015).

For white matter T2, the relaxation time values of the two ROI groups (semioval centrum and cerebellar peduncle) were statistically different (*p* < .0001).

No statistical difference was found between the various age groups in whole‐brain analysis (Figure 4). However, there was a tendency toward longer T2 times with increasing age. We also compared the ROI‐based results with WB results, comparing centrum semiovale values (deemed to be the most representative ones) to those of WB white matter analysis, and insular values to WB gray mater values. When comparing ROI‐based and whole‐brain analysis values among the various age groups, we found differences in T1 and in T2 for both gray and white matter. The heterogeneity of white matter sampled by the WB results could be responsible for the differences between ROI‐based and WB‐based results. These differences were statistically significant in the older age group, suggesting that the heterogeneity of brain parenchyma is more pronounced in older individuals.

In the literature, only a few studies where T2 relaxation time was measured could be identified. Furthermore, they had low number of subjects that were generally younger than our study population (Jiang et al., 2015; Lu et al., 2005). For example, Lu et al. (2005) studied 10 patients (mean age 28 years ± 5). Our values were generally higher than those previously reported (Jiang et al., 2015; Lu et al., 2005). As our data suggest, increasing white matter T2 with age, the difference between our data and those from the literature could be explained by the population in our study being older.

### 4.2 Gray matter

The thalamic T1 was statistically different from insular T1. This difference is not surprising, knowing that the thalamus is less homogenous than gray matter and is composed of several subnuclei (Chakravarty et al., 2006; Fatterpekar et al., 2003). We also found a difference between left and right insula, something that may be explained by the well‐documented asymmetry of the insular cortex (Biduła & Króliczak, 2015; Greve et al., 2013; Vannucci et al., 2019). When the same comparison was made for the thalamus, both sides were far more similar. The T1 results found by the ROI‐based and the WB analyses were statistically different.

Many studies have examined cortical T1 relaxation using various methods and have had different results. Some found high values, like Castro et al. (2010) who reported a mean T1 of 1558 +/− 88 ms using an auto‐segmentation method. Gracien et al. (2017) found a mean T1 of 1649.8 ±68.55 ms for gray matter at baseline and of 1616 ±52.8 ms when reexamining the same subjects seven years later. Although these findings are in agreement with our results, other studies using ROI‐based analyses have reported lower relaxation times, like Lu et al. (2005) and Cho et al. (1997).

Our results of gray matter T1 are in agreement with previous reports. However, the observed discrepancy between ROI‐based and WB analyses is not well documented in the literature. In our data, we can explain this variance by differences in sampling. Indeed, as it is the case for white matter, gray matter RT is also heterogeneous. As with WB methods, much larger regions are captured which are not sampled by the ROI measurements. This hypothesis is supported by the fact that even the right and left insula had different RTs in our study. Furthermore, the observed difference between ROI‐based and WB‐based results seem more pronounced in the older age groups. Indeed, older patients accumulate age‐related changes, which may explain that the parenchymal heterogeneity is greater in this group.

The mean grey matter T2 obtained by the WB analysis was significantly longer than the ROI‐based one. The analysis of gray mater ROI showed significant difference, the mean thalamic value being significantly shorter than that of the insular. When analyzed according to age groups, no statistical difference was found between the various groups in gray matter T2, neither by ROI‐based nor by WB‐based analyses. However, slight differences were found between age groups in the thalamus region, where there was a tendency toward longer T2 increasing with age in WB analysis. As for white matter, it is difficult to find T2 values for the thalamus region in the literature, but our results are consistent with one work by Lu et al. (2005).

Our volumetric analysis showed no statistically significant differences between the age groups except for myelin. However, we could see correlations between age and CSF, myelin status and white matter volume. With increasing age, the amount of white matter and myelin tended to decrease, and the amount of CSF to increase. As the segmentation in SyMR is based on predefined clusters of T1 and T2, this could influence the volumetric analysis. Indeed, the white matter regions containing age‐related alterations (see Figure S3) were excluded by the segmentation and not taken into account in the volumetry. Knowing that relaxation time for tissue changes over the lifespan and that aging induces structural changes in the brain parenchyma, it is very difficult to define a normal range for WM and GM values. In the future, defining a normal range of parenchymal RT could help us diagnose early changes associated with age‐related diseases. One of the most interesting clinical applications of this could be dementia. Indeed, with the ability to define normal aging with MRI, it might be possible to differentiate mild cognitive impairment from dementia.

Despite a relatively large population, the main limitation of our study is the low number of patients in certain subgroups of age. This imbalance between the various groups is certainly decreasing the statistical power of our study. Another limitation is the 2D nature of the acquisition sequence. As there is no 3D SyMRI sequence available in our center, we used a 2D acquisition although we are aware of the signal impairment of a 2D sequence over a 3D one. Future work with 3D SyMRI sequence could address this issue.

## 5 CONCLUSIONS

Synthetic imaging is a new tool that allows for faster imaging, permits to obtain quantitative T1 and T2 and gives the ability to easily perform segmentation of brain parenchyma. As demonstrated, the SyMRI technic can be reliably used in elderly population for parameter estimation. By defining RT values of different brain components of normal‐functioning elderly individuals, this technique may be used as a biomarker for clinical disorders like dementia. However, given the important heterogeneity across the various brain regions suggested by our data, further work and large amount of data is needed to approach the use of the technic as a biomarker.

## CONFLICT OF INTEREST

No conflict of interest has been declared by the authors.

### PEER REVIEW

The peer review history for this article is available at https://publons.com/publon/10.1002/brb3.2449

## Supporting information

SUPPORTING INFORMATION

SUPPORTING INFORMATION

SUPPORTING INFORMATION

SUPPORTING INFORMATION

SUPPORTING INFORMATION

SUPPORTING INFORMATION

## Data Availability

The data that support the findings of this study are available from the corresponding author upon reasonable request.
