# Supplementary figures and images for: Relaxation time of brain tissue in the elderly assessed by synthetic MRI
Source: Brain Behav. 2021 Dec 4;12(1):e2449. doi: 10.1002/brb3.2449 (PMC8785630; doi:10.1002/brb3.2449)

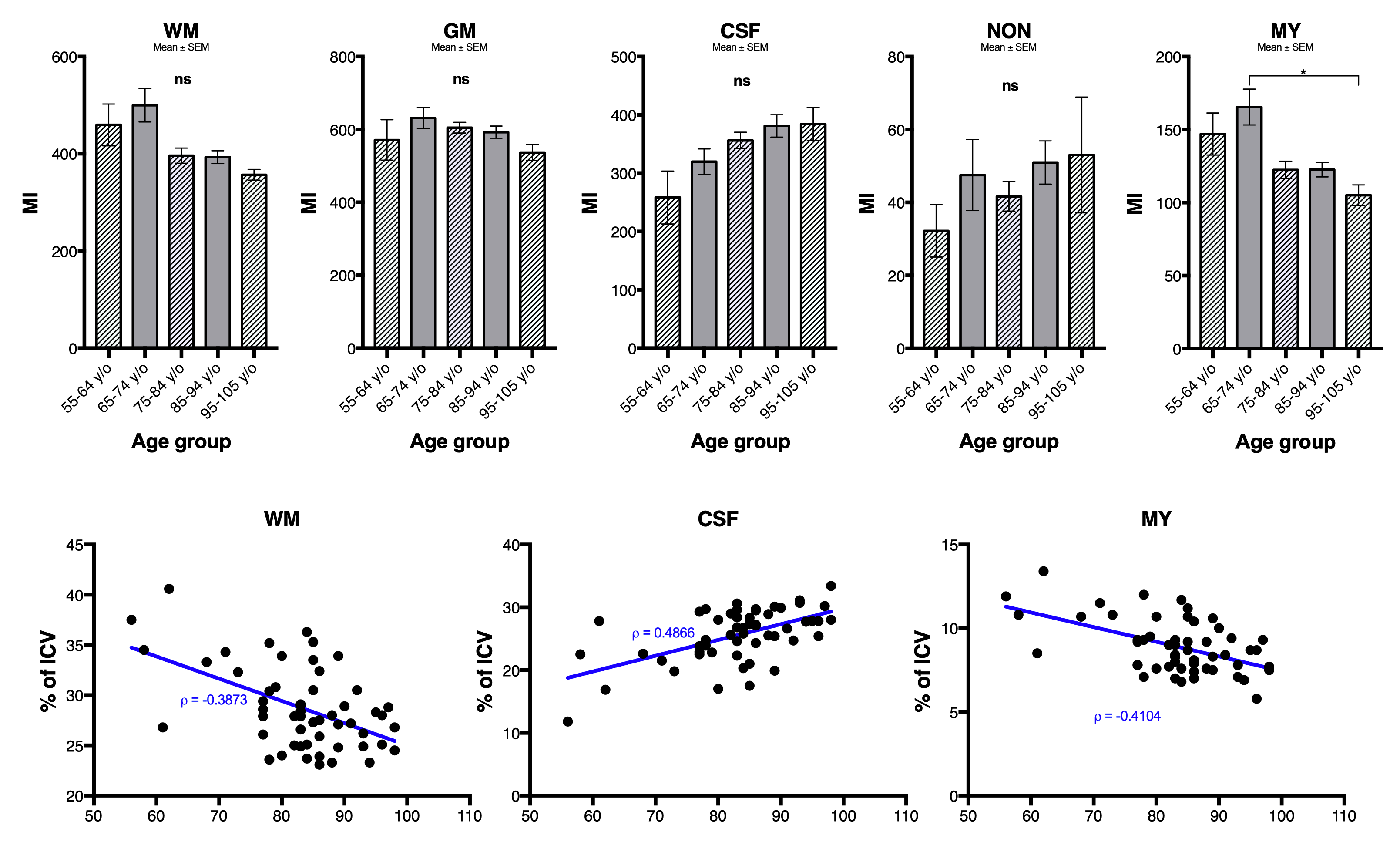

Supplement: Supplementary file 1 — SUPPORTING INFORMATION [file BRB3-12-e2449-s006.tiff]

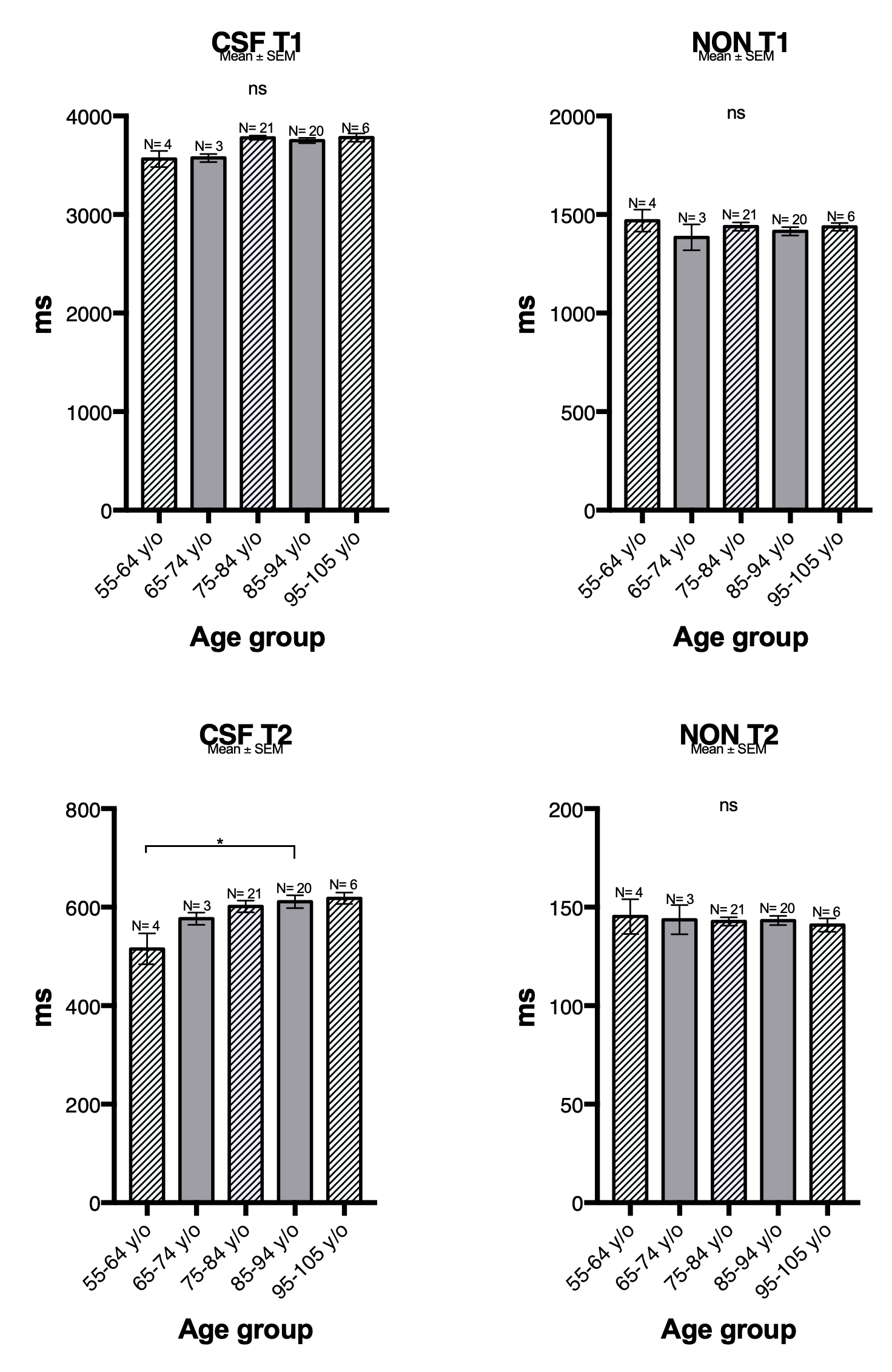

Supplement: Supplementary file 2 — SUPPORTING INFORMATION [file BRB3-12-e2449-s001.tiff]

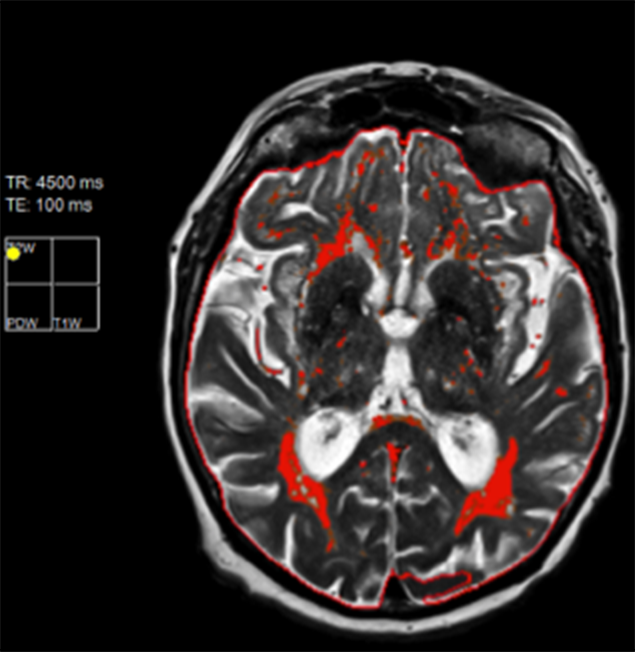

Supplement: Supplementary file 3 — SUPPORTING INFORMATION [file BRB3-12-e2449-s005.tif]

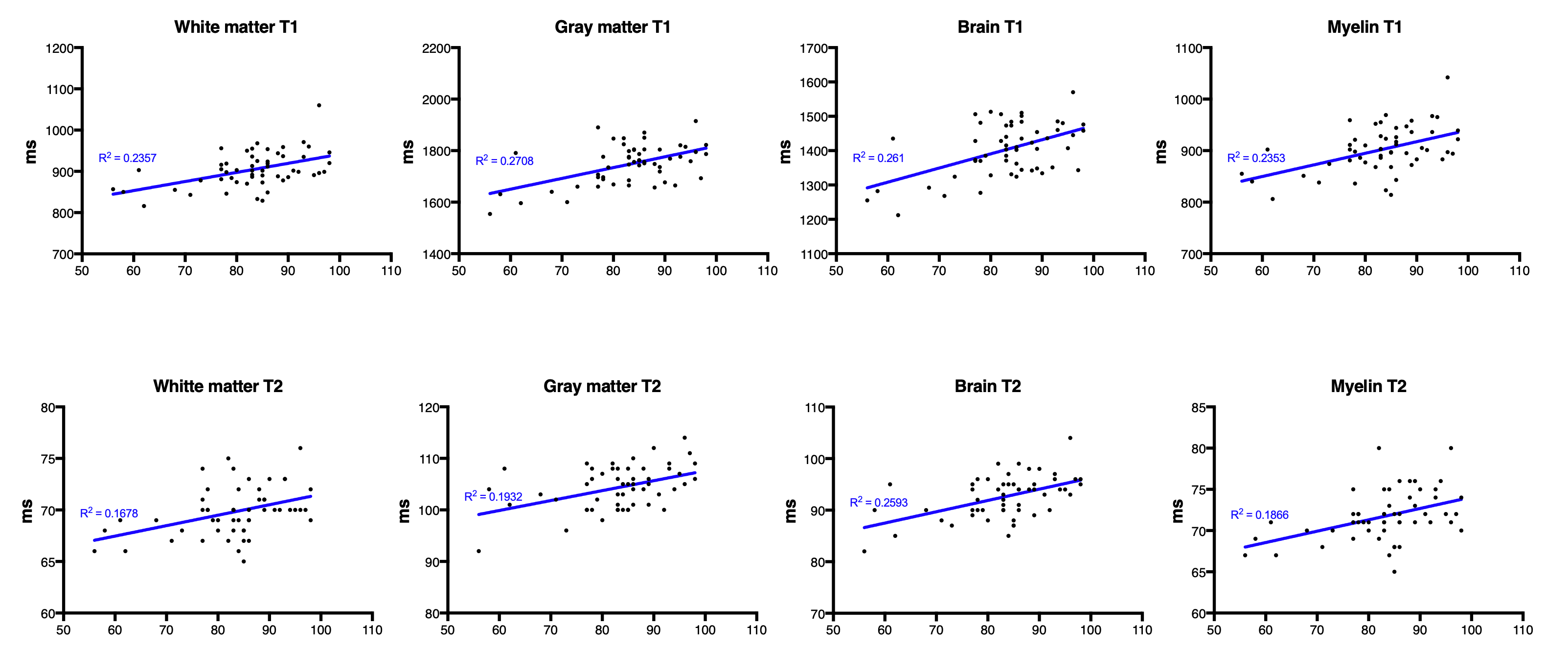

Supplement: Supplementary file 4 — SUPPORTING INFORMATION [file BRB3-12-e2449-s004.tiff]
