# Supplementary material for: Relaxation time of brain tissue in the elderly assessed by synthetic MRI
Source: Brain Behav. 2021 Dec 4;12(1):e2449. doi: 10.1002/brb3.2449 (PMC8785630; doi:10.1002/brb3.2449)
Supplement: Supplementary file 5 — SUPPORTING INFORMATION [file BRB3-12-e2449-s003.pdf]

TABLES

Population characteristic

	N	Female /Male	Mean age (years)
Total population	54	22/32	83
55-64 y/o	4	3/1	59
65-74 y/o	3	0/3	71
75-84 y/o	21	12/9	81
85-94 y/o	20	12/8	88
95-105 y/o	6	5/1	97

Supplementary Table S1: Table showing the general characteristics of our population. From right to left, the columns show the number of patients, the number of female and male and the mean age in year. The upper row represents the whole population included in our study where the followings rows represent the various groups of age in which the patients were pooled.
