# Supplementary material for: Relaxation time of brain tissue in the elderly assessed by synthetic MRI
Source: Brain Behav. 2021 Dec 4;12(1):e2449. doi: 10.1002/brb3.2449 (PMC8785630; doi:10.1002/brb3.2449)
Supplement: Supplementary file 6 — SUPPORTING INFORMATION [file BRB3-12-e2449-s002.pdf]

ROI based Results (ms)

	T1		T2	
	Right	Left	Right	Left
**Gray matter**	1120		74.69	
Thalamus	1027.9	1035.1	68.7	70
Insula	1238	1181	81.35	78.7
**White matter**	900.4		81.84	
Semi-oval centrum	929.2	943.3	104.4	107
Cerebelar peduncle	869.7	859.5	129.3	117.1

Supplementary Table S2: Table showing the mean Region of Interest (ROI)-based results in millisecond across our population. From right to left, the columns show; T1 values for right ROIs, T1 values for left ROIs, T2 values for right and then left ROIs. The upper row shows the mean gray matter results, being the mean between both thalami and both insula. Then from top to bottom, the rows show thalamic, insular values then white matter (mean between both semi-oval centrum and both cerebellar peduncle), semi-oval centrum results and finally the cerebellar peduncle results.
